# Expression Levels of Some Detoxification Genes in Liver and Testis of Rats Exposed to a Single Dose of Methyl-Tertiary Butyl Ether

**DOI:** 10.3889/oamjms.2016.057

**Published:** 2016-06-01

**Authors:** Ahmad Ali Badr, Mostafa Saadat

**Affiliations:** Department of Biology, College of Sciences, Shiraz University, Shiraz 71467-13565, Iran

**Keywords:** Methyl tertiary butyl ether, MTBE, Gene expression, Rat, GST

## Abstract

**AIM:**

Methyl-tertiary-butyl ether (MTBE), a well-known gasoline oxygenate compound, is still used in several countries. Several studies investigated the effects of MTBE on the activity of phase II metabolism enzymes. There is no published data on the effect(s) of short-term exposure to MTBE on mRNA levels of antioxidant genes. Therefore, the present study was carried out.

**METHODS:**

A total of 15 adults male Wistar rats were randomly divided into five equal experimental groups. They received a single dose of 0, 400, 800 and 1600 mg/Kg MTBE in peanut oil by gavages. The final group received no MTBE and peanut oil. After 24 hr animals were slaughtered then livers and testis were removed to extract the total RNA. Real-time PCR was done to detect the gene expressions of glutathione S-transferase family (*Gstt1*, *Gstm1*, and *Gstp1*).

**RESULTS:**

The mRNAs levels of the examined genes neither in liver nor in testis showed a significant difference between the exposed groups and control rats.

**CONCLUSIONS:**

The present data revealed that exposure to a single dose of MTBE has no significant effect on the mRNA levels of the *Gstt1*, *Gstm1*, and *Gstp1* genes.

## Introduction

It has been reported that serum testosterone significantly decreased in male gasoline filling station workers [1]. Methyl-tertiary-butyl ether (MTBE), a well known oxygenated compound which has been used to improve air quality by reducing environmental pollutants, is still used in several Middle East countries. The possible health effects of exposure to gasoline are among important public health issues [2-5]. MTBE is metabolized in liver [3, 6]. There are several studies investigating the effects of MTBE on the activity of phase II metabolism enzymes with inconsistent results [6, 7]. It has been shown that in isolated hepatocytes after treatment with MTBE, glutathione depletion occurred as a consequence of the formation of reactive oxygen species [8].

Glutathione S-transferases (GSTs; EC 2.5.1.18) are a family of enzymes that play an important role in cellular detoxification. The GSTs catalyzing the conjugation of reduced glutathione to a wide range of hydrophobic and electrophilic com-pounds as the first step in detoxification. Using the biochemical, immunological and structural properties of the GSTs, the mammalian cytosolic GSTs are divided into several classes, including mu (Gstm), theta (Gstt), pi (Gstp). Each class is encoded by a single gene or a gene family. It has been reported that the *Gstm1*, *Gstt1*, and *Gstp1* were expressed in many tissues including liver, testis, stomach, brain, etc [http://bioinfo.wilmer.jhu.edu/tiger/]. The specific expression levels of the GSTs are considered to be an adaptive response against the toxicity of endogenous and exogenous metabolites [9-13]. The induced expression of multiple forms of the GSTs appears to be a cellular evolutionary response for protection against chemical toxicity and oxidative stress [14, 15].

Very recently it has been reported that exposure to MTBE was associated with alteration in serum testosterone in experimental rats [7]. It should be noted that the above-mentioned alteration in serum testosterone of filling station workers has been associated with genetic polymorphisms of *GSTT1* and *GSTM1* [1, 16]. Also, it has been reported by our research group that the alterations of end organ markers in residences of Masjid-i-Sulaiman (Khuzestan province, southwest Iran) who are living in contaminating areas and in gasoline filling station workers, were modulated by the genetic polymorphisms of *GSTT1* and *GSTM1* [17-23].

There are only two studies investigating the effect(s) of MTBE on expression levels of GSTs when rats exposed for about one month [24, 25]. We know that in general people are exposed to MTBE for a short period. There is no study on the alterations of mRNA levels of GSTs when rats exposed to a single dose of MTBE. Therefore, the present experiment was carried out.

## Materials and Methods

### Experimental design

A total 15 of adult male Wistar rats (180-200g) were housed in plastic cages under standard animal house conditions with a 12 hr light/dark cycle and a temperature of 25 ± 2°C, received standard pellet food, and tap water was available *ad libitum*. The rats were randomly divided into five equal groups after acclimation period (10 days) which received 0, 400, 800 and 1600 mg/kg MTBE in peanut oil (groundnut oil) by gavages; the final group received no MTBE and peanut oil. After 24 hr, animals were slaughtered and livers and testes were immediately removed and weighted and then were stored at -80°C until use for RNA extraction. MTBE was obtained from Shiraz Oil Refinery (Iran, CAS No. 1634-04-4, 98.8% purity). It should be noted that MTBE is soluble in water maximally (51.26 g/Lit equal to 51 mg/ml). Considering that usually, investigators used higher doses of MTBE [6, 7, 11, 19, 20], therefore, we used oil as a solvent.

This study was approved by Ethics committee of Shiraz University. This work was carried out in accordance with the Code of Ethics of the World Medical Association (Declaration of Helsinki) for experiments involving animal experiments.

### RNA extraction and real-time PCR

Total RNA was extracted from livers and testes by TRIzol method using RNX plus (CinnaGen, Iran). The quality of extracted RNA was assessed by optical density (260/280nm ratios) and the concentration of the RNAs was measured by optical density at 260 nm. All samples had a high quality of RNA (OD_260/280_ = 1.8-2.1). For cDNA synthesis briefly, 500 ng of RNA was reversely transcribed into cDNA according to the cDNA synthesis kit (Takara, Japan) in a final reaction volume of 10 μl using Oligo dT, 1.5x PrimeScript Buffer, Random hexamer and reverse transcriptase enzyme. Real-time quantitative PCR (qPCR) was done to detect the gene expression assay of *Gstm1*, *Gstp1* and *Gstt1* using syber master mix (Amplicon, Germany) on rotor gene 6000 detection system (Corbett life science, Germany). Quantitative real-time PCR conditions were described previously [25]. All primer pairs which used in the present study were designed by Allele ID v7.8 software and they were specific for mRNA, did not amplify genomic DNA, as described previously. Relative differences in gene expression between genotypes were expressed using cycle threshold (Ct) values. ΔCt and ΔΔCt are equal to the difference in threshold cycles (*TBP*, OMIM: 600075; calibrator gene) for target and reference and control and treated samples, respectively. Obtained results of gene expression were analyzed using 2^-∆∆ct^ method [26]. Using this method, the mRNA levels in the untreated normal control group were assumed equal to 1.0 [26].

### Statistical analysis

The fold changes of the *Gstt1*, *Gstm1*, and *Gstp1* mRNA levels were expressed as the mean ± standard error (SE). The significance of the difference between two control sets (not receiving peanut oil and receiving peanut oil) was evaluated with independent two samples *t*-test. Effects of MTBE on the mean of the mRNA levels of the examined genes were investigated using Analysis of Variance (ANOVA) followed by Bonferroni post hoc test. Effects of exposure times of MTBE on means of mRNA levels of the examined genes were assessed using linear regression analysis. Statistical analysis was performed using SPSS statistical software package (version 11.5) for windows (SPSS Inc., Chicago, IL, USA). A two-tailed *P* value < 0.05 is considered to be statistically significant.

## Results and Discussion

Table 1 shows the mean ± SE of the fold changes of the mRNA levels of *Gstt1*, *Gstm1*, and *Gstp1* in liver and testis of the study groups. Effects of MTBE concentrations on the mRNA levels of the examined genes were investigated using Analysis of Variance. Statistical analysis showed that there was no significant difference between the MTBE exposed groups and control rats for the mRNAs levels of examined genes in both liver (For *Gstm1*: F=1.86; df=4,10; P=0.194; For *Gstt1*: F=2.09; df=4,10; P=0.156; For *Gstp1*: F=0.40; df=4,10; P=0.804) and testis (For *Gstm1*: F=0.86; df=4,10; P=0.518; For *Gstt1*: F=1.85; df=4,10; P=0.196; For *Gstp1*: F=0.79; df=4,10; P=0.553).

**Table 1:** Effects of MTBE on the fold changes of the GSTs mRNA levels in liver and testis of rats

Parameters	Control	Peanut oil (Solvent)	MTBE concentration (mg/Kg)		

			400	800	1600
**Liver**					
					
***Gstm1***	1.0	0.94 ± 0.13	1.54 ± 0.14	1.32 ± 0.20	1.62 ± 0.41
***Gstt1***	1.0	0.91 ± 0.17	1.45 ± 0.23	0.94 ± 0.11	0.94 ± 0.16
***Gstp1***	1.0	1.03 ± 0.11	0.90 ± 0.34	0.84 ± 0.39	1.24 ± 0.08
					
**Testis**					
					
***Gstm1***	1.0	0.90 ± 0.13	1.28 ± 0.29	1.13 ± 0.07	1.26 ± 0.21
***Gstt1***	1.0	1.11 ± 0.14	1.44 ± 0.19	1.41 ± 0.21	1.48 ± 0.14
***Gstp1***	1.0	1.32 ± 0.30	1.02 ± 0.04	1.24 ± 0.30	1.41 ± 0.16

**Note:** Data were expressed as mean ± SE.

The present data confirm previously published articles which they reported that exposure to MTBE was not associated with alteration in the GSTs gene expression levels [24, 25]. Based on the several studies, it is suggested that MTBE may be attributed to induction of oxidative stress in the reproductive system [26, 27]. However, our present data indicating that effect of MTBE on reproductive systems is not via alteration in mRNA levels of GSTs.

It should be noted that in filling station workers, which were occupationally exposed to gasoline, alteration in serum testosterone was significantly modulated by the *GSTT1* and *GSTM1* polymorphisms [1, 16]. Also, it has been reported that the *GSTT1* and *GSTM1* polymorphisms may modulate the alterations in several end organ markers (such as liver function indies, sex hormones, etc) in persons exposed to natural sour gas [17-23].

Previously we reported the mRNA levels of *Gstt1*, *Gstm1*, and *Gstp1* in the liver of rats exposed to various concentrations of MTBE when the animals were treated for 30 days [25]. In order to neutralize the effect(s) of concentrations of MTBE and usage of peanut oil on the relationship between exposure time and mRNA levels of the examined genes, multiple linear regression analysis were used. Statistical analysis revealed that in livers of the treated rats, the mRNA levels of the *Gstm1* (standardized coefficient β = -0.513, t = 3.63, P = 0.001) and *Gstt1* (standardized coefficient β = -0.632, t = 2.34, P = 0.025) significantly associated with exposure time. This means that the mRNA levels of *Gstt1* and *Gstm1* initially increased at 24 hr and then decreased at 30 days. Very recently, a similar pattern was reported for other mRNA levels of other genes involved in cellular detoxification after human SH-SY5Y cells exposed to methadone and morphine [29, 30]. There is no significant relationship between the levels of *Gstp1* with exposure time in livers of the treated rats (standardized coefficient β = -0.155, t = 0.96, P = 0.341).

The present study revealed that mRNA levels of *Gstt1* and *Gstm1* did not show significant deviation from their control levels (Table 1). As mentioned in our previous report [23], the difference between filling station workers and the present study, at least in part might be interpreted by this point that gasoline contains several components, whereas MTBE is a pure material. Other experiments should be carried out to explain the possible effect(s) of MTBE on end reproductive system and liver function tests; and as well as the relationship between alterations in expression levels of antioxidant genes and activities of antioxidant enzymes after treatment with MTBE.

## References

[1] Saadat M, Monzavi N (2008). Genetic polymorphisms of glutathione S-transferase T1 (GSTT1) and alterations of sex hormones in filling-station workers. Fertil Steril.

[2] Prah J, Ashley D, Blount B (2004). Dermal, oral and inhalation pharmacokinetics of methyl tertiary butyl ether (MTBE) in human volunteers. Toxicol Sci.

[3] Phillips S, Palmer RB, Brody A (2008). Epidemiology, toxicokinetics, and health effects of methyl tert-butyl ether (MTBE). J Med Toxicol.

[4] Hu D, Yang J, Liu Y (2016). Health risk assessment for inhalation exposure to methyl tertiary butyl ether at petrol stations in southern China. Int J Environ Res Public Health.

[5] Zhang L, Qin J, Zhang Z (2016). Concentrations and potential health risks of methyl tertiary-butyl ether (MTBE) in air and drinking water from Nanning, South China. Sci Total Environ.

[6] Elovaara E, Stockmann-Juvala H, Mikkola JV (2007). Interactive effects of methyl tertiary-butyl ether (MTBE) and tertiary-amyl methyl ether (TAME), ethanol and some drugs: Triglyceridemia, liver toxicity, and induction of CYP (2E1, 2B1) and phase II enzymes in female Wistar rats. Environ Toxicol Pharmacol.

[7] Khalili L, Gholami S, Ansari-Lari M (2015). Evaluation of offspring sex ratio, sex hormones and antioxidant enzymes following exposure to methyl tertiary butyl ether in adult male Sprague-Dawley rats. EXCLI J.

[8] Salimi A, Vaghar-Moussavi M, Seydi E (2016). Toxicity of methyl tertiary-butyl ether on human blood lymphocytes. Environ Sci Pollut Res Int.

[9] Hayes JD, Flanagan JU, Jowsey IR (2005). Glutathione transferases. Annu Rev Pharmacol Toxicol.

[10] Li W, Ichihara G, Wang H (2010). Change of gonad gene expression profile in male F344 rats after exposure to 1-bromopropane. Wei Sheng Yan Jiu.

[11] Sherratt PJ, Manson MM, Thomson AM (1998). Increased bioactivation of dihaloalkanes in rat liver due to induction of class theta glutathione S-transferase T1-1. Biochem J.

[12] Nteeba J, Ganesan S, Keating AF (2014). Impact of obesity on ovotoxicity induced by 7,12-dimethylbenz[a]anthracene in mice. Biol Reprod.

[13] Sharma A, Saurabh K, Yadav S (2013). Expression profiling of selected genes of toxication and detoxication pathways in peripheral blood lymphocytes as a biomarker for predicting toxicity of environmental chemicals. Int J Hyg Environ Health.

[14] Shimada Y, Dewa Y, Ichimura R (2010). Antioxidant enzymatically modified isoquercitrin suppresses the development of liver preneoplastic lesions in rats induced by beta-naphthoflavone. Toxicology.

[15] Raza H (2011). Dual localization of glutathione S-transferase in the cytosol and mitochondria: implications in oxidative stress, toxicity and disease. FEBS J.

[16] Saadat M, Bahaoddini S, Saadat I (2013). Alteration of serum sex hormonal profile in male gasoline filling station workers in respect to their polymorphism of glutathione S-transferase M1. Environ Toxicol Pharmacol.

[17] Ansari-Lari M, Saadat M, Hadi N (2003). Modulation of hematology changes by polymorphism of glutathione S-transferase M1 and T1. Biochem Biophys Res Commun.

[18] Ansari-Lari M, Saadat M, Hadi N (2004). Influence of GSTT1 null genotype on the offspring sex ratio of gasoline filling station workers. J Epidemiol Community Health.

[19] Saadat M (2004). Genetic polymorphisms of glutathione S-transferases M1 and T1 modulate hematological changes of individuals chronically exposed to natural sour gas. Biochem Biophys Res Commun.

[20] Saadat M, Bahaoddini A, Mohabatkar H (2004). Polymorphisms of glutathione S-transferase M1 and T1 modulate blood pressure of individuals chronically exposed to natural sour gas containing sulfur compounds. Biochem Biophys Res Commun.

[21] Saadat M, Ansari-Lari M (2005). Alterations of liver function test indices of filling station workers with respect of genetic polymorphisms of GSTM1 and GSTT1. Cancer Lett.

[22] Saadat M, Zendeh-Boodi Z (2008). Association between genetic polymorphism of GSTT1 and depression score in individuals chronically exposed to natural sour gas. Neurosci Lett.

[23] Zendeh-Boodi Z, Saadat M (2008). Genetic polymorphism of GSTT1 may be under natural selection in a population chronically exposed to natural sour gas. Mol Biol Rep.

[24] Zhou W, Huang G, Zhang H (1999). Effect of methyl tertiary butyl ether on the expression of proto-oncogenes and function genes. Wei Sheng Yan Jiu.

[25] Badr AA, Saadat I, Saadat M (2015). Study of liver function and expression of some detoxification genes in the male rats exposed to methyl-tertiary butyl ether. Egypt J Med Hum Genet.

[26] Schmittgen TD, Livak KJ (2008). Analyzing real-time PCR data by the comparative CT method. Nat Protoc.

[27] Li D, Liu Q, Gong Y (2009). Cytotoxicity and oxidative stress study in cultured rat sertoli cells with methyl tert-butyl ether (MTBE) exposure. Reprod Toxicol.

[28] Li D, Yuan C, Gong Y (2008). The effects of methyl tert-butyl ether (MTBE) on the male rat reproductive system. Food Chem Toxicol.

[29] Saify K, Saadat M (2015). Expression patterns of antioxidant genes in human SH-SY5Y cells after treatment with methadone. Psychiatry Res.

[30] Saify K, Saadat I, Saadat M (2015). Down-regulation of antioxidant genes in human SH-SY5Y cells after treatment with morphine. Life Sci.

